# Derivation and Validation of a Prognostic Scoring Model Based on Clinical and Pathological Features for Risk Stratification in Oral Squamous Cell Carcinoma Patients: A Retrospective Multicenter Study

**DOI:** 10.3389/fonc.2021.652553

**Published:** 2021-05-28

**Authors:** Jiaying Zhou, Huan Li, Bin Cheng, Ruoyan Cao, Fengyuan Zou, Dong Yang, Xiang Liu, Ming Song, Tong Wu

**Affiliations:** ^1^ Hospital of Stomatology, Guanghua School of Stomatology, Sun Yat-sen University, Guangzhou, China; ^2^ Guangdong Provincial Key Laboratory of Stomatology, Guanghua School of Stomatology, Sun Yat-sen University, Guangzhou, China; ^3^ Department of ICU, Sun Yat-sen University Cancer Center, State Key Laboratory of Oncology in South China, Collaborative Innovation Center for Cancer Medicine, Guangzhou, China; ^4^ Department of Data Sciences, AID Cloud Technology Co., Ltd, Guangzhou, China; ^5^ Department of Head and Neck Surgery, Sun Yat-sen University Cancer Center, State Key Laboratory of Oncology in South China, Collaborative Innovation Center for Cancer Medicine, Guangzhou, China

**Keywords:** oral squamous cell carcinoma, prediction model, prognosis, risk stratification, nomogram

## Abstract

**Objective:**

To develop and validate a simple-to-use prognostic scoring model based on clinical and pathological features which can predict overall survival (OS) of patients with oral squamous cell carcinoma (OSCC) and facilitate personalized treatment planning.

**Materials and Methods:**

OSCC patients (n = 404) from a public hospital were divided into a training cohort (n = 282) and an internal validation cohort (n = 122). A total of 12 clinical and pathological features were included in Kaplan–Meier analysis to identify the factors associated with OS. Multivariable Cox proportional hazards regression analysis was performed to further identify important variables and establish prognostic models. Nomogram was generated to predict the individual’s 1-, 3- and 5-year OS rates. The performance of the prognostic scoring model was compared with that of the pathological one and the AJCC TNM staging system by the receiver operating characteristic curve (ROC), concordance index (C-index), calibration curve, and decision curve analysis (DCA). Patients were classified into high- and low-risk groups according to the risk scores of the nomogram. The nomogram-illustrated model was independently tested in an external validation cohort of 95 patients.

**Results:**

Four significant variables (physical examination-tumor size, imaging examination-tumor size, pathological nodal involvement stage, and histologic grade) were included into the nomogram-illustrated model (clinical–pathological model). The area under the ROC curve (AUC) of the clinical–pathological model was 0.687, 0.719, and 0.722 for 1-, 3- and 5-year survival, respectively, which was superior to that of the pathological model (AUC = 0.649, 0.707, 0.717, respectively) and AJCC TNM staging system (AUC = 0.628, 0.668, 0.677, respectively). The clinical–pathological model exhibited improved discriminative power compared with pathological model and AJCC TNM staging system (C-index = 0.755, 0.702, 0.642, respectively) in the external validation cohort. The calibration curves and DCA also displayed excellent predictive performances.

**Conclusion:**

This clinical and pathological feature based prognostic scoring model showed better predictive ability compared with the pathological one, which would be a useful tool of personalized accurate risk stratification and precision therapy planning for OSCC patients.

## Introduction

Prognostic prediction models are widely utilized both in clinic and research to estimate the probability that a certain outcome will occur within a specific time period in an individual (1). A reliable prognostic model is essential in individual risk quantification and stratification, which is fundamental in personalized treatment plan development. Furthermore, it can also help to provide a basis for health economic assessment of cost-effectiveness (2).

Recent global estimates have revealed 377,713 new cases and 177,757 deaths of oral cancer in 2020 (3). Oral squamous cell carcinoma (OSCC) is the most common oral cancer, accounting for more than 90% of all oral cancers (4). Although surgical resection remains the primary treatment at present, more and more therapeutic options such as radiotherapy, chemotherapy and immunotherapy have emerged. Advances in treatments improved the quality of life and life expectancy of patients. However, the 5-year overall survival rate of OSCC patients was still less than 60% (5). Therefore, how to assess the prognostic risk and choose the most suitable treatment for individuals is challenging for clinicians (6).

The most commonly and widely used prognostic model for oral cancer is based on the American Joint Committee on Cancer (AJCC) tumor, lymph node, and metastases (TNM) staging system (7). However, the pathological TNM stage does not allow a comprehensive assessment for the prognosis prediction of patients. Many other risk factors, including age, smoking status, primary site, and clinical examination results, should be considered for individualized prognosis (8). A growing number of tumor molecular biomarker models have been highlighted for their potential predictive abilities (9). But more and more studies demonstrate that due to the methodological heterogeneity, biomarker testing lacks sufficient accuracy, which is difficult to define specific biomarkers for OSCC prognosis prediction (10, 11).

In this study, we aimed to develop a prognostic scoring model using the widely available physical and imaging data, as well as the pathological data to predict 1-, 3- and 5-year OS in OSCC patients after surgery. The model would help the clinician to customize adjuvant treatment program in addition to surgical resection. We combined the most relevant prognosticators into nomogram, which could help clinicians to define the risk profile of individual patient intuitively and effectively. Furthermore, the model was validated in an external patient cohort. This model will not only contribute to provide a more accurate OSCC prognosis, but also help to facilitate personalized treatment planning.

## Materials and Methods

### Patient Selection

In this multicenter retrospective study, we firstly collected 4,089 OSCC patients from the Head and Neck Surgery Department of the Sun Yat-sen University Cancer Center (SYSUCC, Guangdong, China). The inclusion criteria of patients were as follows: (1) received pretreatment clinical assessment including tumor size and nodal involvement of physical and imaging examinations, (2) received curative-intent surgical resection, (3) received postoperative pathological confirmation, (4) follow-up time greater than 6 months. The patients were excluded according to the following criteria: (1) patients experienced distant metastasis at the time of diagnosis, (2) patients with previous history of OSCC, (3) subgroup with small sample size. After applying the criteria, 404 patients from SYSUCC between 2000 and 2016 were enrolled in this study and randomly split into the training cohort (n = 282) and internal validation cohort (n = 122) with 7/3 split ratio.

**Figure 1:**
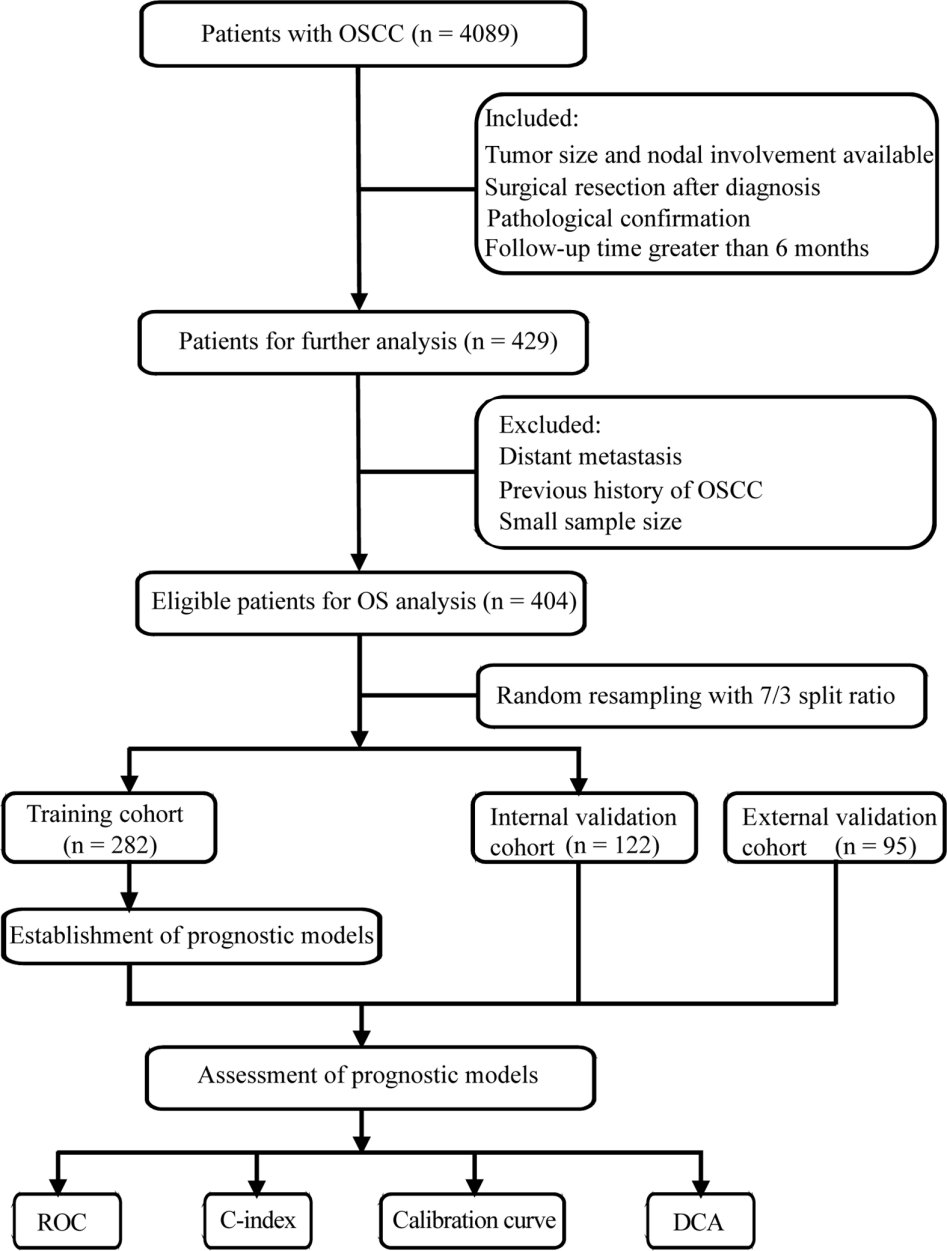
Flow chart of screening process and experimental procedure. OSCC, oral squamous cell carcinoma; ROC, receiver operating characteristic curve; C-index, concordance index; DCA, decision curve analysis.

OSCC patients satisfying the aforementioned inclusion and exclusion criteria were also obtained from the Hospital of Stomatology of the Sun Yat-sen University between 2013 and 2018 (Guangdong, China). In total, 95 patients were designated as the external validation cohort. The screening process are shown in **Figure 1**. All research procedures were approved by the Ethics Committee of the Sun Yat-sen University Cancer Center and Hospital of Stomatology of the Sun Yat-sen University. Informed consents for data collection and analysis were obtained from patients.

### Variable Enrollment

A total of 12 key variables were categorized into three data types for each OSCC case. Social demographic data included gender, age, radiotherapy history for head and neck cancer, and smoking history. Clinical data included primary site, physical examination-tumor size (PE-T), imaging examination-tumor size (IE-T), physical examination-nodal involvement (PE-N), and imaging examination-nodal involvement (IE-N). Imaging examination only included CT and MRI in our study. Pathological data included pathological tumor stage (P-T), pathological nodal involvement stage (P-N), and histologic grade. All the data were summarized in **Table 1**. The clinical and pathological TN stage were classified according to the 7^th^ edition of the AJCC staging system for oral cancer. The endpoint of this study was overall survival (OS), which referred to the time interval from surgery to death or the last follow-up (12). The survival time of patients who were still alive at the last date of follow-up was given as censored data.

**Table 1:** Characteristics description in the training and validation cohorts.

Characteristics	Subtype	Train cohort (n = 282)	Internal validation cohort (n = 122)	*p*-value	External validation cohort (n = 95)	*p*-value*
Gender	Male Female	192 (68.09%) 90 (31.91%)	87 (71.31%) 35 (28.69%)	1	64 (67.37%) 31 (32.63%)	1
Age	<60 ≥60	181 (64.18%) 101 (35.82%)	74 (60.66%) 48 (39.34%)	1	64 (67.37%) 31 (32.63%)	1
Radiotherapy history	No/Unknown Yes	271 (96.1%) 11 (3.90%)	118 (96.72%) 4 (3.28%)	1	95 (100%) 0 (0%)	NA
Smoking history	No Yes	150 (53.19%) 132 (46.81%)	71 (58.2%) 51 (41.8%)	0.974	57 (60.0%) 38 (40.0%)	0.870
Primary tumor site	Tongue Floor of mouth Gingiva Hard palate Others	142 (50.35%) 28 (9.93%) 58 (20.57%) 33 (11.7%) 21 (7.45%)	71 (58.2%) 8 (6.56%) 17 (13.93%) 18 (14.75%) 8 (6.56%)	0.636	63 (66.31%) 9 (9.47%) 10 (10.53%) 2 (2.11%) 11 (11.58%)	0.047
PE-T	(0-2] cm (2-4] cm >4 cm	70 (24.82%) 138 (48.94%) 74 (26.24%)	23 (18.85%) 78 (63.93%) 21 (17.21%)	0.459	24 (25.26%) 63 (66.32%) 8 (8.42%)	0.019
IE-T	(0-2] cm (2-4] cm >4 cm	85 (30.14%) 147 (52.13%) 50 (17.73%)	39 (31.97%) 69 (56.56%) 14 (11.48%)	0.867	37 (38.95%) 50 (52.63%) 8 (8.42%)	0.532
PE-N	N0 N1 N2	164 (58.16%) 71 (25.18%) 47 (16.67%)	70 (57.38%) 27 (22.13%) 25 (20.49%)	1	60 (63.16%) 31 (32.63%) 4 (4.21%)	0.199
IE-N	N0 N1 N2	197 (69.86%) 39 (13.83%) 46 (16.31%)	81 (66.39%) 17 (13.93%) 24 (19.67%)	1	30 (31.58%) 57 (60.0%) 8 (8.42%)	<0.001
P-T	T1 T2 T3 T4	68 (24.11%) 115 (40.78%) 57 (20.21%) 42 (14.89%)	23 (18.85%) 70 (57.38%) 11 (9.02%) 18 (14.75%)	0.204	18 (18.95%) 49 (51.58%) 11 (11.58%) 17 (17.89%)	0.966
P-N	N0 N1 N2	173 (61.35%) 65 (23.05%) 44 (15.6%)	74 (60.66%) 25 (20.49%) 23 (18.85%)	0.999	68 (71.58%) 10 (10.53%) 17 (17.89%)	0.413
Histologic grade	Well differentiated Moderately differentiated Poorly differentiated	178 (63.12%) 76 (26.95%) 28 (9.93%)	66 (54.1%) 49 (40.16%) 7 (5.74%)	0.459	47 (49.47%) 46 (48.42%) 2 (2.11%)	0.127
AJCC TNM stage	I II III IV	54(19.15%) 72(25.53%) 80(28.37%) 76(26.95%)	17(13.93%) 42(34.43%) 28(22.95%) 35(28.69%)	0.963	16(16.84%) 40(42.11%) 12(12.63%) 27(28.42%)	0.099
Follow-up months	Median (range)	25.6 (11-71)	29.4 (12-93)	0.222	42 (8-69)	0.184

*Compared with the training cohort. PE-T, physical examination-tumor size; IE-T, imaging examination-tumor size; PE-N, physical examination-nodal involvement; IE-N, imaging examination-nodal involvement; P-T, pathological tumor stage; P-N, pathological nodal involvement stage; NA, not applicable.

### Statistical Analysis

Kaplan–Meier analysis was used to estimate OS and detect intersections between the variables. The independent prognostic factors affecting OS were identified by univariate and multivariate Cox regression analyses. The qualified prognostic factors with significant differences in the univariate analysis were incorporated into the multivariate analysis. Stepwise regression was adopted to remove the non-significant factors, which ensured each variable in the resulting independent variable subset was significant to the dependent variable and the remaining variables were not multicollinear. The results were shown as hazard ratios (HR) with 95% confidence interval (CI). Then, an integrated nomogram was established to predict 1-, 3-, 5-year OS based on multivariate Cox proportional hazards regression model. Finally, the clinical–pathological model was validated internally and externally according to the TRIPOD statement (13).

The performances of the prognostic model were evaluated by various methods, involving the time-dependent receiver operating characteristic (ROC) curve and the value of the area under the ROC curve (AUC), Harrell’s concordance index (C-index), calibration curve, and decision curve analysis (DCA). ROC was used to assess the sensitivity and specificity of the model. C-index was determined to evaluate the model’s discriminative power between the predicted model and actual chance of experiencing the events (14). 1,000 bootstrap resamples were used to obtain the intervals of the C-index. The purpose of the calibration curve is to evaluate the agreement between the predictive values and observation values in the probabilities of 3- and 5-year survival of individuals. DCA was used to determine the net benefit of using the prognostic model at various threshold probabilities, which would be helpful to evaluate the actual needs for clinical decision-making (15). The total risk points of each patient were calculated according to the established nomogram. An optimal cut-off point was determined by the R package “maxstat” in the training cohort to classify patients as high-risk and low-risk groups.

All analyses were conducted using Python version 3.7.1 and R version 4.0.0 (R Foundation for Statistical Computing, Vienna, Austria). All statistical tests were two-sided with statistical significance defined as a p < 0.05. The Kolmogorov–Smirnov test was used to compare the distribution in the training and validation cohorts between different subgroups. Kaplan–Meier analysis was performed, and log-rank tests were used to determine the significance of the survival differences. In addition, Hosmer–Lemeshow tests were applied to evaluate the goodness-of-fit of the calibration curve, p > 0.05 represented good calibration (16).

## Results

### Patient Characteristics

The demographic and clinicopathologic characteristics of the training and validation cohorts are summarized in **Table 1**. Of the 282 individuals in the training cohort, 68.09% of the patients were male, and patients over 60 years accounted for 35.82%. Only minority of patients (3.9%) had a radiotherapy history for head and neck cancer, and about half of patients (46.81%) had a smoking history. The 95 individuals in the external validation cohort were slightly younger with a lower prevalence of smoking. The median period of follow-up of the training cohort was shorter than that of the external validation cohort (25.6 vs. 42 months). The tongue was the most common primary tumor site in both the training and validation cohorts (50.35, 58.2, 66.31%, respectively). There was no statistically significant difference in the distribution of the features between the training and internal validation cohorts (p > 0.05), while the distribution of the primary tumor site, PE-T, and IE-N between the training and external validation cohorts was significantly different (p < 0.05) (**Table 1**).

### Screening Independent Prognostic Factors

**Figure 2:**
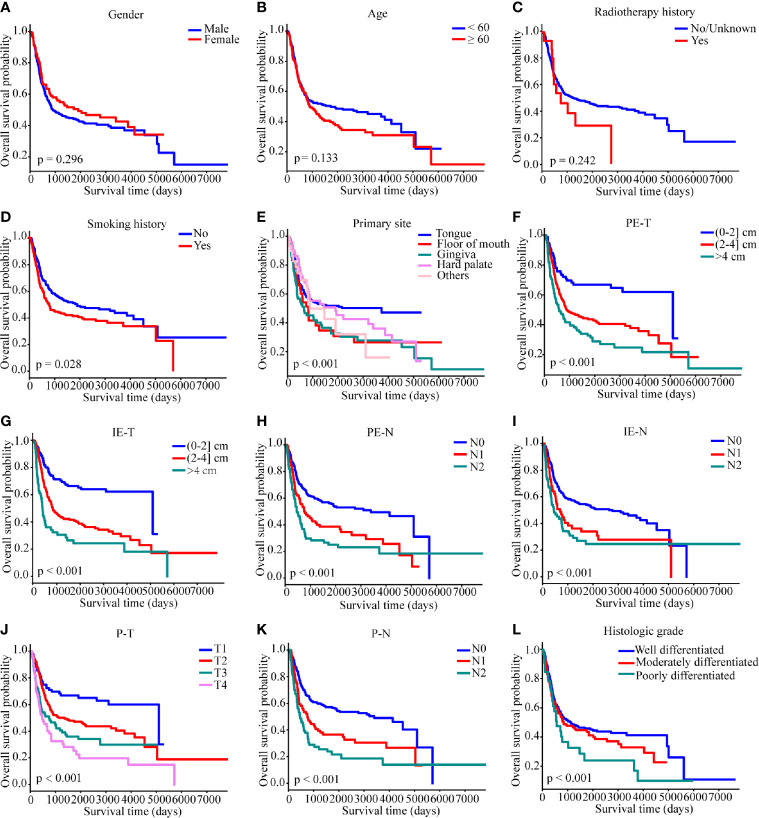
Kaplan–Meier survival curves of overall survival in the SYSUCC cohort. **(A)** gender, **(B)** age, **(C)** radiotherapy history, **(D)** smoking history, **(E)** primary site, **(F)** PE-T, **(G)** IE-T, **(H)** PE-N, **(I)** IE-N, **(J)** P-T, **(K)** P-N, **(L)** histologic grade. Survival curves were compared by the log-rank test, and *p <*0.05 was considered as statistically significant. PE-T, physical examination-tumor size; IE-T, imaging examination-tumor size; PE-N, physical examination-nodal involvement; IE-N, imaging examination-nodal involvement; P-T, pathological tumor stage; P-N, pathological nodal involvement stage; T, tumor; N, lymph node.

Kaplan–Meier analysis showed that smoking history, primary site, PE-T, IE-T, PE-N, IE-N, P-T, P-N, and histologic grade were significantly associated with OS (all p < 0.05), while gender, age, and radiotherapy history displayed non-significance (p > 0.05) (**Figure 2**). All the available characteristics were included in the univariate analysis. There were statistically significant survival differences in the characteristics of primary tumor site, PE-T, IE-T, PE-N, IE-N, P-T, P-N, histologic grade in the univariate analysis (**Table 2**). These significant variables in the Kaplan–Meier curves and the univariate analysis were included in the multivariate analysis to further screen out significant factors. In the multivariable stepwise regression analysis, PE-T (HR = 3.811; 95% CI, 1.210–12.004; p = 0.022), IE-T (HR = 4.135; 95% CI, 1.343–12.736; p = 0.013), P-N (HR = 1.834; 95% CI, 1.241–2.710; p = 0.002), and histologic grade (HR, 1.649; 95% CI, 1.083–2.511; p = 0.02) were significantly associated with OS in the training cohort (**Table 2**). Meanwhile, P-T, P-N, and histologic grade were significantly related to the outcome in the pathological model (p < 0.05, **Supplementary Table S1**).

**Table 2:** Univariate and multivariate Cox analysis for the clinical-pathological model.

Characteristics	Univariate analysis	*p*−value	Multivariate analysis	*p*−value
	HR (95% CI)		HR (95% CI)	
Gender	0.862 (0.621–1.195)	0.373		
Age	1.182 (0.869–1.609)	0.286		
Radiotherapy history	1.596 (0.824–3.093)	0.166		
Smoking history	1.287 (0.954–1.736)	0.099		
Site— tongue	Reference			
Site —Floor of mouth	1.758 (1.086–2.846)	0.022		
Site—Gingiva	1.702 (1.171–2.472)	0.005		
Site—Hard palate	1.192 (0.752–1.890)	0.454		
Site—Others	0.916 (0.464–1.807)	0.800		
PE-T	1.285 (1.170–1.411)	<0.001	3.811 (1.210–12.004)	0.022
IE-T	1.301 (1.183–1.432)	<0.001	4.135 (1.343–12.736)	0.013
PE-N	1.490 (1.234–1.800)	<0.001		
IE-N	1.408 (1.171–1.693)	<0.001		
P-T	1.389 (1.196–1.612)	<0.001		
P-N	1.548 (1.282–1.870)	<0.001	1.834 (1.241–2.710)	0.002
Histologic grade	1.290 (1.043–1.596)	0.019	1.649 (1.083–2.511)	0.020

HR, hazard ratio; CI, confidence interval; other abbreviations as in **Table 1**.

### Development of the Prognostic Model and Nomogram

**Figure 3:**
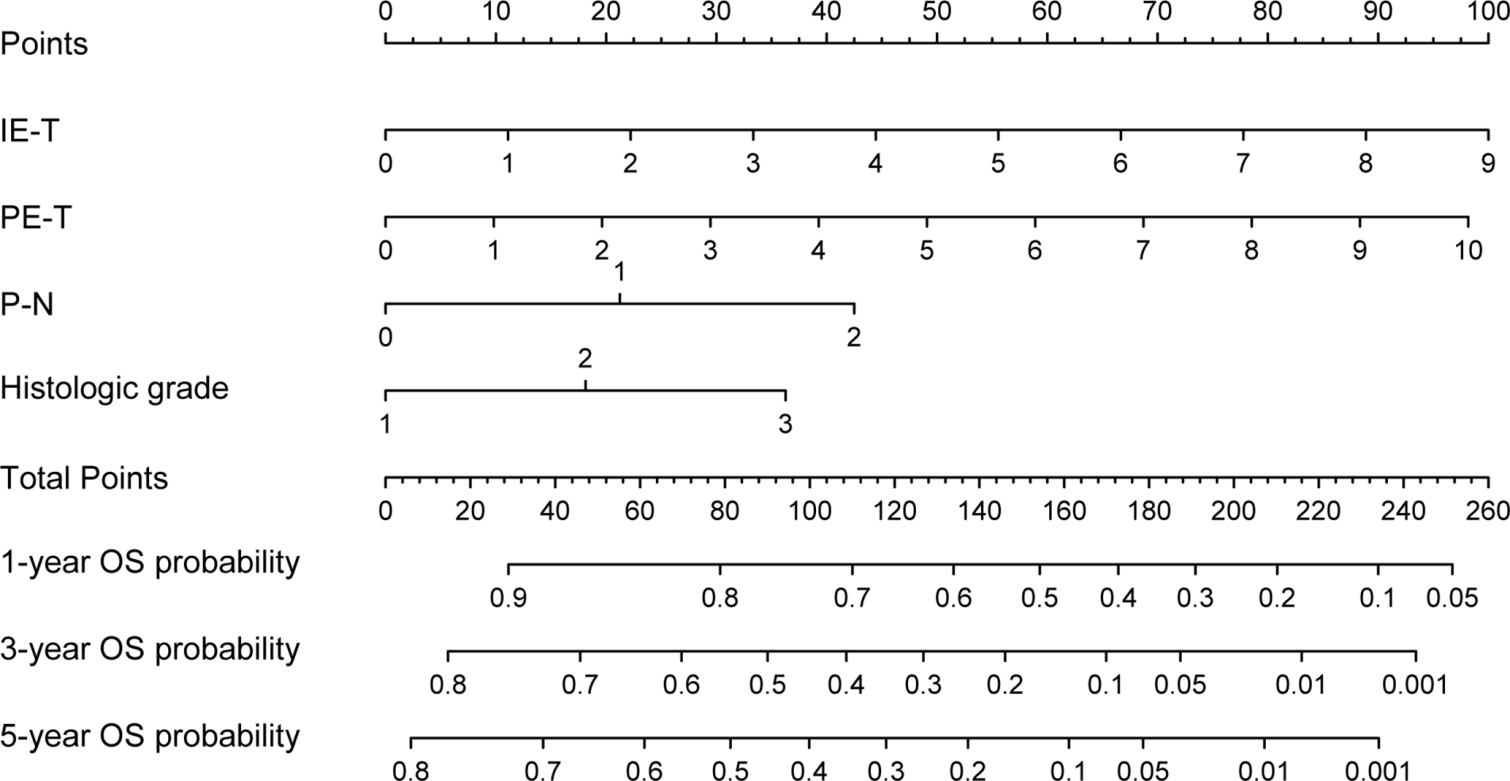
Nomogram based on the clinical-pathological model for the prediction of 1-, 3- and 5-year OS. PE-T, physical examination-tumor size; IE-T, imaging examination-tumor size; P-N, pathological nodal involvement stage; OS, overall survival.

Based on the results of the multivariable Cox regression model, four independent variables were incorporated to develop a more accurate nomogram for optimizing personalized prognostic assessment and predicting 1-, 3- and 5-year OS (**Figure 3**). Higher score was associated with a poor prognosis. The nomogram of the pathological model was shown in **Supplementary Figure S1**.

### Performance and Validation of the Prognostic Model

**Figure 4:**
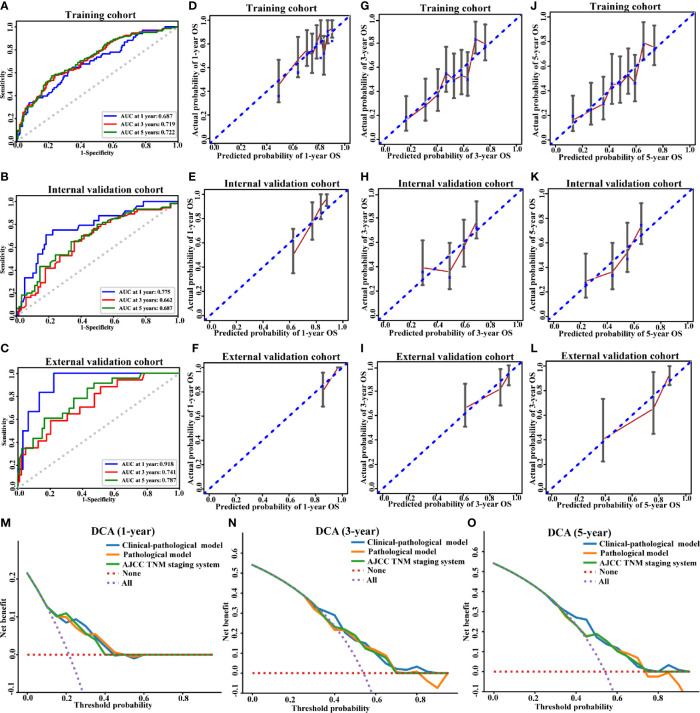
Performance of the clinical-pathological model. ROC curve **(A–C)**, calibration curves for 1-year OS **(D–F)**, calibration curves for 3-year OS **(G–I)**, calibration curves for 5-year OS **(J–L)**, and decision curve analysis **(M–O)** for the training **(A, D, G, J)**, internal validation **(B, E, H, K, M–O)** and external validation cohorts **(C, F, I, L)**. AUC, area under the curve; OS, overall survival.

The predictive accuracy between the clinical-pathological model and the pathological model has been compared. In the training cohort, the 1-, 3-, and 5-year AUC values of the clinical–pathological model for OS prediction were 0.687, 0.719, 0.722 (**Figure 4A**), which were superior to those of the pathological one (0.649, 0.707, 0.717, respectively) and AJCC TNM staging system (0.628, 0.668, 0.677, respectively), demonstrating excellent sensitivity and specificity for the clinical–pathological model (**Supplementary Figures S2A**, **S3A**). Similarly, the 1-, 3-, and 5-year AUCs in the internal validation cohort (0.775, 0.662, 0.687, respectively; **Figure 4B**) and external validation cohort (0.918, 0.741, 0.787, respectively; **Figure 4C**) of the clinical–pathological model also showed better discriminative ability compared with the pathological one and the AJCC TNM staging system (**Supplementary Figures S2B, C**, **S3B, C**).

The C-indices of the clinical–pathological model displayed better predictive performance than that of the pathological model in the training (0.664; 95% CI, 0.615–0.711 vs. 0.638; 95% CI, 0.592–0.683), internal validation (0.679; 95% CI, 0.609–0.75 vs. 0.655; 95% CI, 0.578–0.726) and external validation cohorts (0.755; 95% CI, 0.644–0.853 vs. 0.702; 95% CI, 0.621–0.778) (**Table 3**). The clinical–pathological model exhibited superior discriminative power for OS prediction compared with the pathological model and the AJCC TNM staging system.

**Table 3:** The C-indices for prediction of overall survival.

Model	Training cohort		Internal validation cohort		External validation cohort	
	C-index	95% CI	C-index	95% CI	C-index	95% CI
Clinical—pathological model	0.664	0.615–0.711	0.679	0.609–0.750	0.755	0.644–0.853
Pathological model	0.638	0.592–0.683	0.655	0.578–0.726	0.702	0.621–0.778
AJCC TNM staging system	0.610	0.564–0.653	0.660	0.594–0.720	0.642	0.567–0.713

C-index, concordance index; CI, confidence interval.

The calibration curves of 1-, 3- and 5-year OS and non-significant Hosmer–Lemeshow test demonstrated a good agreement between the prediction and observation values (p > 0.05) in the training (**Figures 4D, G, J**) and validation cohorts (**Figures 4E, F, H, I, K, L**), indicating that there was no deviation from the perfect fit. The calibration curves of pathological model were shown in **Supplementary Figures S2D–L**.

DCA analysis was conducted to evaluate the clinical value of our model. In the validation cohort, for predicted threshold probability between 30 and 70%, both the clinical–pathological and pathological models showed a positive net benefit for 3- and 5-year OS. Furthermore, the clinical–pathological model had a better net benefit for decision-making with the threshold probability within 30 and 50% illustrated by DCA (**Figures 4N, O**). Collectively, the threshold probabilities of the clinical–pathological model had better net benefits for predicting the 1-, 3- and 5-year OS in OSCC patients compared with the pathological one and the AJCC TNM staging system (**Figures 4M–O**).

### Risk Stratification Based on the Nomogram

**Figure 5:**
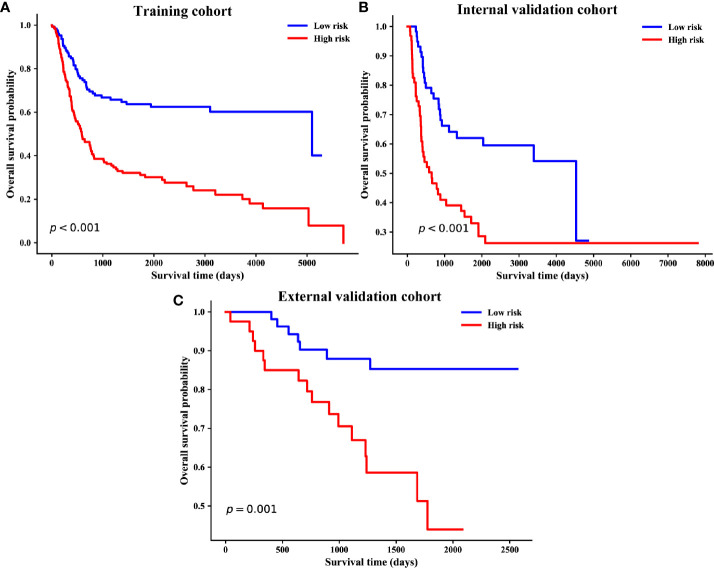
Kaplan–Meier survival curves of overall survival based on risk score of the clinical–pathological nomogram in the training **(A)**, internal validation **(B)** and external validation cohorts **(C)**.

Based on the individualized risk points of the nomogram, patients were divided into low-risk and high-risk groups in the training, internal, and external validation cohorts (**Figures 5A–C** **)**. The optimal cut-off value was 79.08 for the clinical–pathological nomogram, and the Kaplan–Meier curves revealed that the high-risk group (total points > 79.08) was significantly correlated with a poor prognosis. The optimal point effectively distinguished populations of low-risk and high-risk, demonstrating a good prognostic classification for OSCC patients.

## Discussion

Reliable prognostic factors are indispensable for properly stratifying the risk of the individual patient and avoid unnecessary overtreatment as well as unjustified toxicity. Clinical prognostic judgement of OSCC mainly focuses on the most classical AJCC TNM staging system (17–19). Besides the traditional pathological criterion, the multiple biomarkers detection such as protein-coding genes, messenger RNAs, and non-coding RNAs in body fluids (such as saliva and serum) and tumor tissues have gained much attention in prognosis prediction (20–23). However, traditional pathological TNM staging system only considered the anatomical extent of the disease without considering the nonanatomic factors, which can’t fully reflect the accurate prognosis (24). A variety of clinicopathological parameters like age, gender, as well as clinical and pathological features of the tumor were also associated with the prognosis of OSCC patients (8, 25, 26). The single characteristic is usually insufficient to predict individual survival, while a combination can provide better prognostic reliability (27). Due to the lack of exact independent predictable biomarker, current biomarker testing was limited in the practical application (28).

Current treatment strategies for OSCC vary from radical surgery and radiotherapy to chemotherapy and molecular targeted therapy (29). Therapeutic effectiveness, health care costs, and personal affordability all will have influence on treatment process (30). From the perspective of patients and clinicians, the prognosis judgement would be of great importance in postoperative risk stratification and personalizing selection of adjuvant treatment for OSCC patients who underwent surgical resection (31). To develop a simple-to-use prediction model, we combined the clinical variables with the pathological TN stage, taking the individual differences in clinical examination into account. The illustrated-nomogram model finally suggested that integrating the preoperative data of physical and imaging examination with pathological data may be a comprehensive, economical and convenient method for clinicians to predict the prognosis of OSCC patients.

Nomograms have been frequently used for cancer prognosis prediction via a simple visualization modality. In our study, a visualized nomogram encompassed clinical and pathological risk factors that were easy to obtain and routinely collected was developed. Through the intuitive nomogram, the interrelation between variables and outcome was demonstrated and the probability of outcome events could be easily calculated by clinicians. Each subtype within these covariates was assigned a point on the point scale. Adding the points together of each variable was able to calculate a total point. The clinician would get the prediction probability of 1-, 3- and 5-year OS by locating the total point on the bottom scales. In our model, individualized score for each patient was calculated according to the nomogram, and the patients were successfully divided into low- and high-risk groups. The two groups showed significantly different prognosis. For high-risk patients, the traditional surgical resection cannot achieve satisfactory outcome, so alternative adjuvant treatment could be considered for the postoperative therapeutic scheme.

In the clinical–pathological model, the four variables including IE-T, PE-T, P-N, and histologic grade were significantly related to the outcome. Data of tumor size in physical examination were acquired by clinicians. Due to the specific location of oral cancer, clinicians could directly observe and measure the extent of the tumor, which ensured the relative accuracy and repeatability of our data (32). As an indispensable tool for clinical decision, physical examination still plays a significant role in the prognostic risk assessment in our study.

Tumor size in imaging examination also played a crucial part in our prognostic model. As a vital part of precision medicine, imaging examination has been proven its utility in identifying the multi-dimensional shape and location characteristics of a tumor (33). In 2017, tumor depth of invasion (DOI) was introduced into the 8^th^ edition AJCC staging system. The AJCC 8^th^ manual suggested that DOI could be reliably defined by the preoperative imaging (34). Weimar et al. successfully used the measurements of tumor thickness as a modifier for T stage in 8^th^ AJCC based on the preoperative imaging examination such as computed tomography (CT) and magnetic resonance imaging (MRI) (35). At present, radiomics have been proven in identifying the shape and location characteristics of the tumor (36). However, radiomics require professional quantitative extraction of high-dimensional mineable data from all types of medical images, which mainly relies on professional radiologist for analysis (37). For most clinical surgeons, it’s difficult to complete the complex radiomics analysis and make a prognosis judgement. Therefore, in our research, we only used the simple tumor size and nodal involvement stage obtained by imaging examination. Especially, the tumor size incorporated into the nomogram was analyzed as a continuous variable, which is convenient for clinicians to operate.

Although the background characteristics were different between independent hospitals, our nomogram-illustrated model still showed strong predictive ability in the external validation cohort, indicating our model could be widely applied to predict OS. Remarkably, early postoperative adjuvant therapy may be appropriate for patients considered at high risk for OS, such as those with high nomogram points. However, in the current analysis, the imaging examination data mainly included CT and MRI data. To gain further evidence and confirmation, large-scale prospective study and more up-to-date data from different equipment are needed to validate the generalization.

## Conclusion

Collectively, a new nomogram-illustrated model was developed and validated for the OSCC patients without distant metastases from retrospective data. The tumor size of physical and imaging examination, pathological nodal involvement stage, and histologic grade were significantly associated with OS. Our clinical–pathological model was accessible and practical, which showed improved discriminatory ability relative to the pathological model. The clinical–pathological model might act as an effective method to improve the individualized prognostic evaluation through patient-specific characteristics, which may help to optimize postoperative therapeutic strategies.

## Data Availability Statement

The original contributions presented in the study are included in the article/**Supplementary Material**. Further inquiries can be directed to the corresponding authors.

## Ethics Statement

Written informed consent was obtained from the individual(s) for the publication of any potentially identifiable images or data included in this article.

## Author Contributions

JZ and TW were involved in the design and conception. HL, RC, FZ, and DY conducted the acquisition of data, statistical analysis, and interpretation of data. JZ, HL, and TW drafted the paper. BC, MS, and XL retrieved the relevant literatures and revised the paper. All authors contributed to the article and approved the submitted version.

## Funding

This work was supported by the National Natural Science Foundation of China (No. 81600878) and the key project of National Natural Science Foundation of China (No. 81630025).

## Conflict of Interest

Authors FZ, DY, and XL were employed by the company AID Cloud Technology Co., Ltd.

The remaining authors declare that the research was conducted in the absence of any commercial or financial relationship that could be construed as a potential conflict of interest.

## Supplementary Material

Supplementary Figure 1 Nomogram based on the pathological model for the prediction of 1-, 3- and 5-year OS. P-T, pathological tumor stage; P-N, pathological nodal involvement stage; OS, overall survival.

Supplementary Figure 2 Performance of the pathological model. ROC curve **(A–C)**, calibration curves for 1-year OS **(D–F)**, calibration curves for 3-year OS **(G–I)**, and calibration curves for 5-year OS **(J–L)** for the training **(A, D, G, J)**, internal validation **(B, E, H, K)** and external validation cohorts **(C, F, I, L)**.

Supplementary Figure 3 The ROC curves based on the AJCC TNM staging system for the prediction of 1-, 3- and 5-year OS in the training **(A)**, internal validation **(B)**, and external validation cohorts **(C)**.
