# Supplementary figures and images for: Derivation and Validation of a Prognostic Scoring Model Based on Clinical and Pathological Features for Risk Stratification in Oral Squamous Cell Carcinoma Patients: A Retrospective Multicenter Study
Source: Front Oncol. 2021 May 28;11:652553. doi: 10.3389/fonc.2021.652553 (PMC8195273; doi:10.3389/fonc.2021.652553)

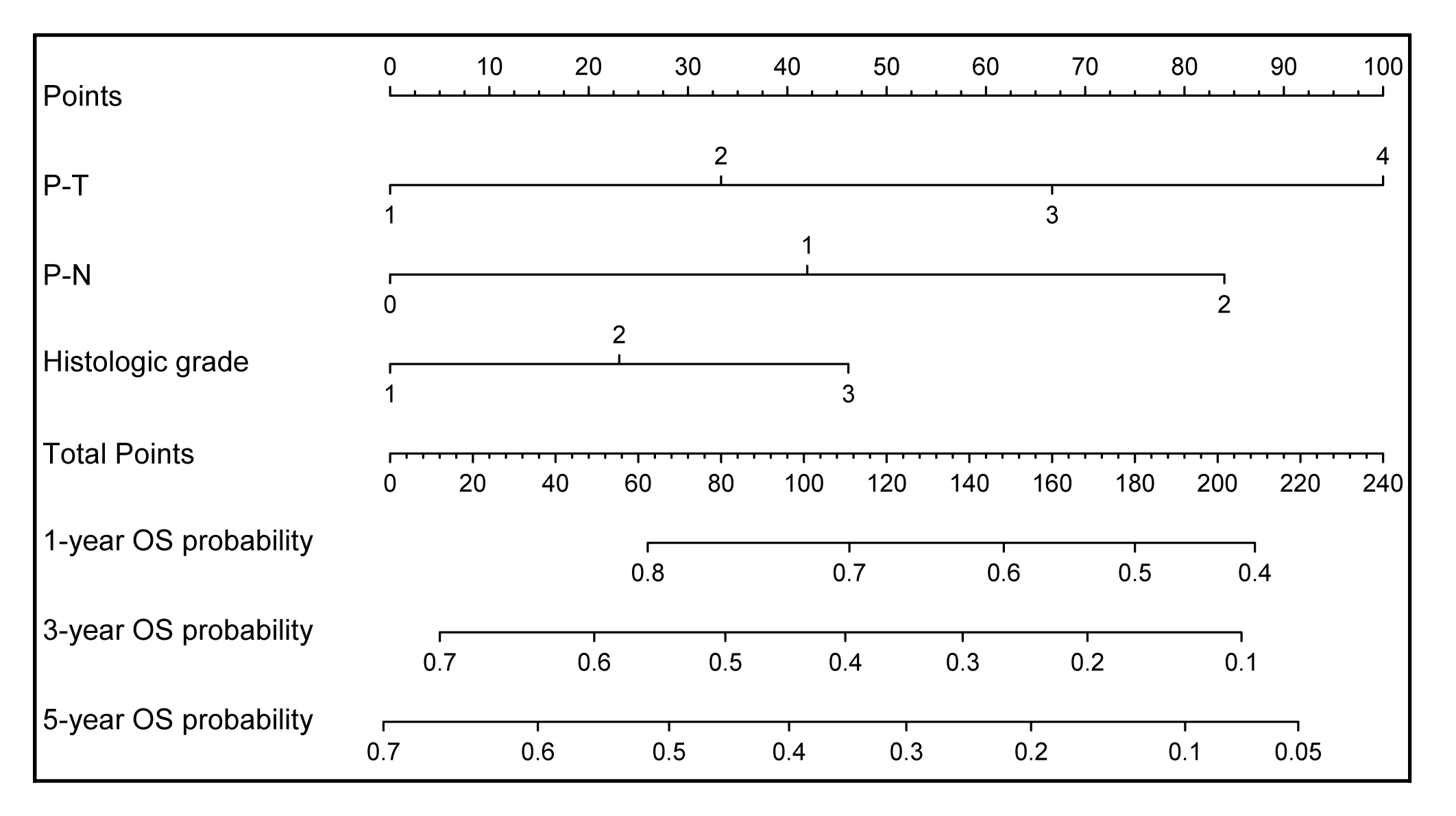

Supplement: Supplementary Figure 1 — Nomogram based on the pathological model for the prediction of 1-, 3- and 5-year OS. P-T, pathological tumor stage; P-N, pathological nodal involvement stage; OS, overall survival. [file Image_1.tif]

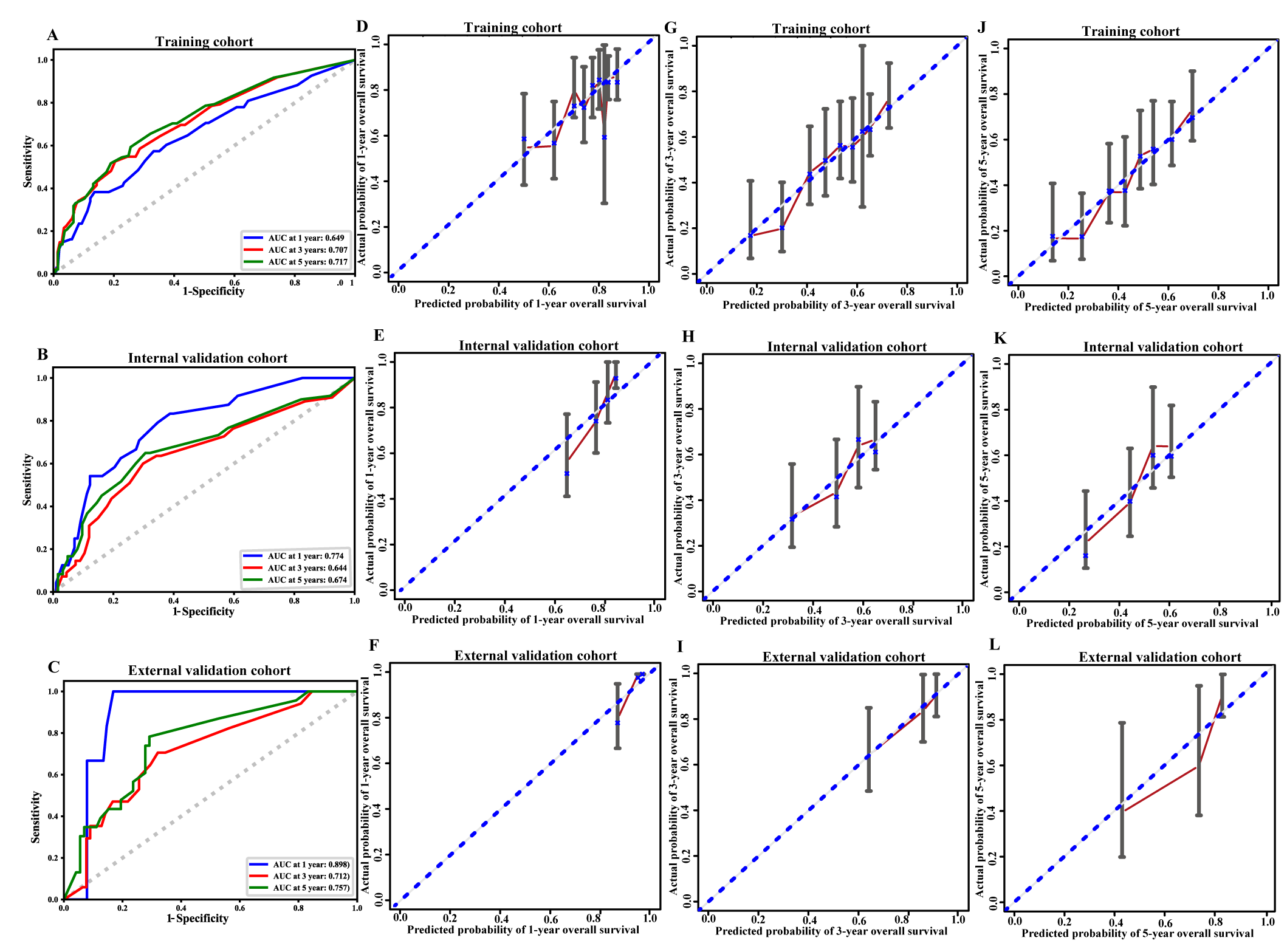

Supplement: Supplementary Figure 2 — Performance of the pathological model. ROC curve (A–C), calibration curves for 1-year OS (D–F), calibration curves for 3-year OS (G–I), and calibration curves for 5-year OS (J–L) for the training (A, D, G, J), internal validation (B, E, H, K) and external validation cohorts (C, F, I, L). [file Image_2.tif]

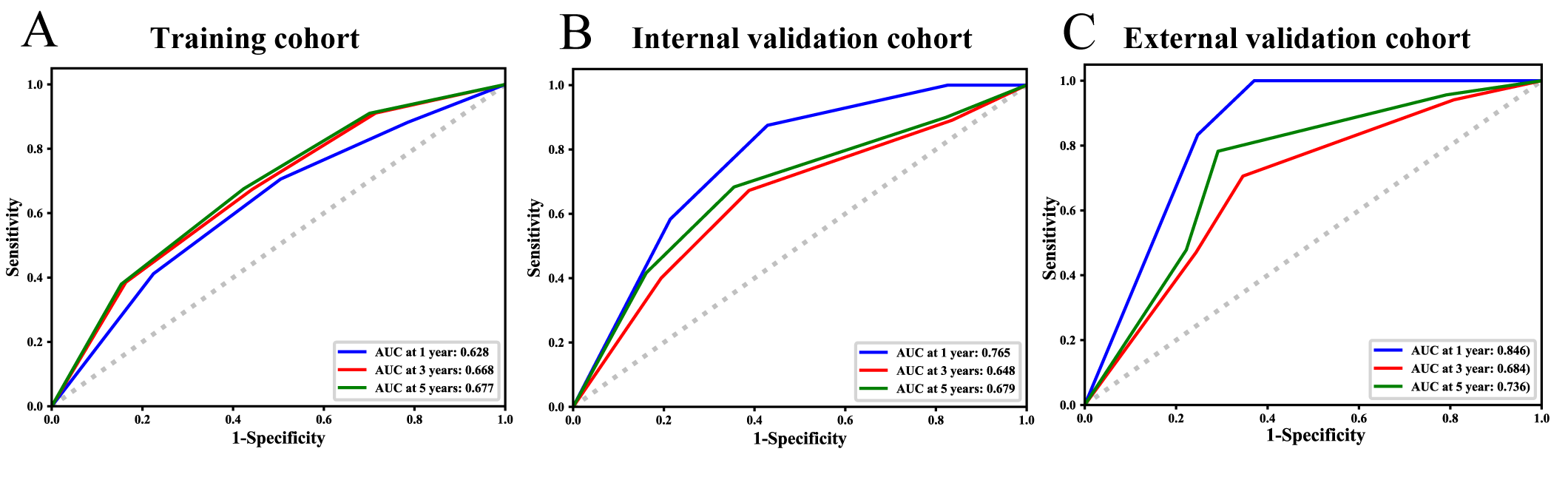

Supplement: Supplementary Figure 3 — The ROC curves based on the AJCC TNM staging system for the prediction of 1-, 3- and 5-year OS in the training (A), internal validation (B), and external validation cohorts (C). [file Image_3.tif]
