# Supplementary material for: Derivation and Validation of a Prognostic Scoring Model Based on Clinical and Pathological Features for Risk Stratification in Oral Squamous Cell Carcinoma Patients: A Retrospective Multicenter Study
Source: Front Oncol. 2021 May 28;11:652553. doi: 10.3389/fonc.2021.652553 (PMC8195273; doi:10.3389/fonc.2021.652553)
Supplement: Supplementary file 4 [file Table_1.docx]

**Supplementary Table S1** Univariate and multivariate Cox analysis for the pathological model

| Characteristics | Univariate analysis |  | Multivariate analysis |  |
| --- | --- | --- | --- | --- |
|  | HR (95% CI) | *p*‑value | HR (95% CI) | *p*‑value |
| Gender | 0.862 (0.621-1.195) | 0.373 |  |  |
| Age | 1.182 (0.869-1.609) | 0.286 |  |  |
| Radiotherapy history | 1.596 (0.824-3.093) | 0.166 |  |  |
| Smoking history | 1.287 (0.954-1.736) | 0.099 |  |  |
| Site - tongue | reference |  |  |  |
| Site - Floor of mouth | 1.758 (1.086-2.846) | 0.022 |  |  |
| Site - Gingiva | 1.702 (1.171-2.472) | 0.005 |  |  |
| Site - Hard palate | 1.192 (0.752-1.890) | 0.454 |  |  |
| Site - Others | 0.916 (0.464-1.807) | 0.800 |  |  |
| P-T | 1.389 (1.196-1.612) | <0.001 | 2.425 (1.537-3.828) | <0.001 |
| P-N | 1.548 (1.282-1.870) | <0.001 | 2.126 (1.448-3.121) | <0.001 |
| Histologic grade | 1.290 (1.043-1.596) | 0.019 | 1.517 (1.000-2.302) | 0.049 |

*Abbr*: P-T, pathological tumor stage; P-N, pathological nodal involvement stage; HR, hazard ratio; CI, confidence interval.
